# Some Results in the Treatment of Epilepsy

**Published:** 1893-11

**Authors:** 


SOME RESULTS IN THE TREATMENT OF EPILEPSY.—In a paper read before the Michigan State Medical Society, Dr. David Inglis announces the following conclusions: "To sum up: I believe that the routine use of bromides does serious harm. That is a serious mistake to go doggedly on with bromides, in any case in which the attacks of grand mal, but more especially in petit mal, persist or increase in frequency while the patient is taking bromides.

"I believe that bromides should be given in full doses to begin with; that, if they are to prove of benefit in a given case, the good effect will be promptly shown. The dose should then be diminished, and always carefully watched. Failure of memory, mental torpor, change of character, and worse things than an occasional explosion, and when the toxic effects of the drug fiast appear it ought to be stopped at once. I believe that we have, in antifebrin and its analogues, a group of remedies which form efficient substitutes for the bromides. They can be given for long periods with marked benefit, and their use is without any deleterious effect upon the mental state. This alone gives them an immense advantage over the bromedes. One precaution, however, must be observed; the drugs need not be given in large doses, but there are persons on whose circulatory apparatus even moderate doses exercise a depressing effect. Such cases are not fit for the antifebrin treatment.—*The Medical Bulletin.*

